# Insulin resistance and uric acid-related metabolic disturbances jointly identify individuals at high risk of advanced cardiovascular-kidney-metabolic syndrome: a population-based study

**DOI:** 10.3389/fnut.2026.1933816

**Published:** 2026-09-03

**Authors:** Shengzhu Huang, Jingwei Lin, Lingyu Ye, Fuxiang Fang, Yezhou Wu, Xuefang Liang, Junwu Ran, Zhuokun Wei, Fuhua Hu, Yibi Lan, Zheng Wen, Mingli Li, Chao Wang, Yu Peng, Zengnan Mo

**Affiliations:** 1 Center for Genomic and Personalized Medicine, Guangxi key Laboratory for Genomic and Personalized Medicine, Guangxi Collaborative Innovation Center for Genomic and Personalized Medicine, Guangxi Medical University, Nanning, Guangxi, China; 2 Institute of Urology and Nephrology, First Affiliated Hospital of Guangxi Medical University, Guangxi Medical University, Nanning, Guangxi, China; 3 School of Life Sciences and Medical Engineering, Guangxi Medical University, Nanning, Guangxi, China; 4 Bobai County People's Hospital, Yulin, Guangxi, China

**Keywords:** cardiovascular-kidney-metabolic syndrome, metabolic biomarkers, metabolic dysfunction, serum uric acid, triglyceride-glucose index

## Abstract

**Background:**

Cardiovascular-kidney-metabolic (CKM) syndrome encompasses metabolic abnormalities, chronic kidney disease, and cardiovascular disease. The triglyceride–glucose (TyG) index reflects insulin resistance-related glucose–lipid imbalance, whereas serum uric acid (SUA) reflects purine metabolism and renal urate handling. However, their independent and joint associations with advanced CKM remain unclear.

**Methods:**

We conducted a cross-sectional analysis of 8,939 adults from the Hakka Biobank baseline survey. Advanced CKM (stages 3–4) was defined according to the American Heart Association framework. Multivariable logistic regression examined associations of TyG and SUA with advanced CKM. Restricted cubic splines assessed nonlinearity, and joint associations used the same-dataset ROC-derived TyG threshold of 8.89 and sex-specific hyperuricemia (HUA) thresholds. Sensitivity and subgroup analyses assessed robustness and heterogeneity, while AUC and continuous net reclassification improvement (NRI) evaluated in-sample model performance.

**Results:**

Among 8,939 participants, 1,164 had advanced CKM. After adjustment, each 1-unit increase in TyG was associated with greater odds of advanced CKM (OR, 1.81; 95% CI, 1.64–2.01), as was each standard deviation increase in SUA (OR, 1.38; 95% CI, 1.28–1.48). TyG showed an approximately linear positive relationship, whereas SUA showed a J-shaped relationship (*P* for nonlinearity = 0.004), which was more evident in women. Participants with both high TyG and HUA had the greatest odds (OR, 3.01; 95% CI, 2.49–3.64). Results remained consistent across six sensitivity analyses involving medication adjustment, exclusions for renal impairment or metabolic medication use, and menopausal-status adjustment among women. Jointly adding TyG and SUA improved in-sample discrimination (AUC, 0.816 vs. 0.797; *p* < 0.001) and reclassification (NRI, 0.433; 95% CI, 0.372–0.496). The combined association was stronger in women (*P* for interaction = 0.028).

**Conclusion:**

Higher TyG and SUA levels, particularly their co-occurrence, were associated with prevalent advanced CKM. These biomarkers may characterize distinct but overlapping metabolic profiles relevant to CKM assessment. Their potential value for community-based evaluation requires confirmation in prospective studies with external validation; no causal or treatment implications can be inferred from these cross-sectional findings.

## Introduction

Cardiovascular-kidney-metabolic (CKM) syndrome has recently emerged as an integrated framework linking metabolic dysfunction, cardiovascular disease, and chronic kidney disease (CKD) along a shared pathobiological continuum (1). Rather than representing isolated comorbidities, these conditions interact through interconnected metabolic, renal, and vascular pathways that drive multiorgan dysfunction and excess cardiovascular risk (2, 3). This concept highlights the need for simple and scalable biomarkers that can capture upstream biological vulnerability and improve identification of individuals at risk of advanced CKM.

Insulin resistance is a central mechanism in CKM, but direct assessment remains impractical in routine care and large-scale studies (4–7). The triglyceride-glucose (TyG) index has therefore gained attention as an accessible surrogate of insulin resistance and has been associated with a wide range of adverse cardiometabolic outcomes (8–11). Serum uric acid (SUA), in contrast, may reflect a partly distinct dimension of CKM biology related to oxidative stress, endothelial dysfunction, and renal urate handling (12–14). Importantly, urate metabolism and insulin resistance are biologically interconnected (15, 16), suggesting that TyG and SUA may capture related but non-identical aspects of CKM vulnerability.

However, current evidence remains fragmented. Most prior studies have evaluated TyG or SUA separately in relation to individual cardiometabolic or renal outcomes, whereas few have examined them jointly within an explicit CKM framework. Whether these markers differ in their dose–response associations with CKM, whether their coexistence identifies a particularly high-risk phenotype, and whether their combined assessment improves risk stratification beyond conventional factors remain unclear. Therefore, we investigated the independent, dose–response, and joint associations of TyG and SUA with advanced CKM in a community-based Chinese adult population and further evaluated their incremental discriminatory value and potential sex-specific differences.

## Data source and study population

### Study design and population

**Figure 1:**
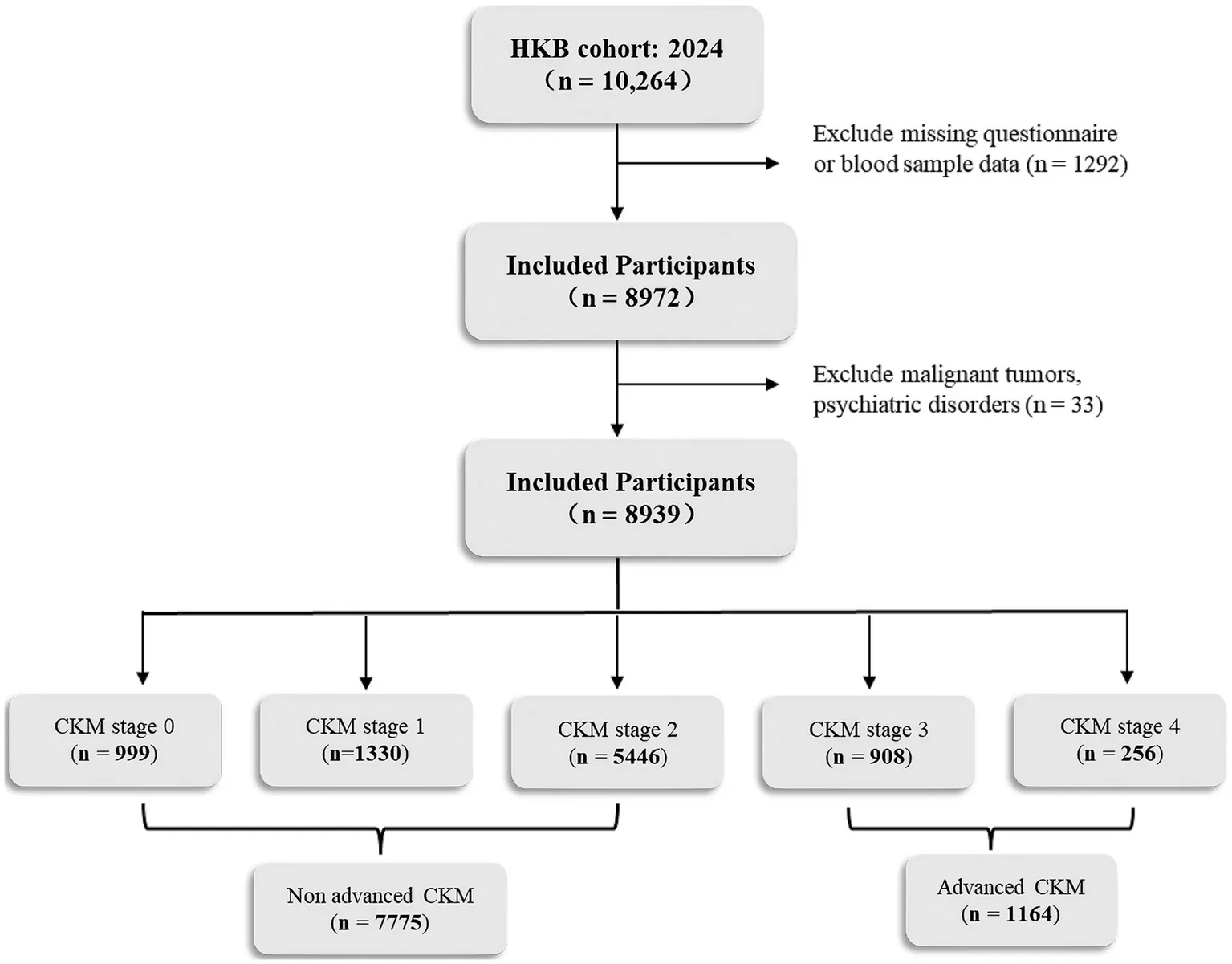
Participants include screening flowcharts.

To address these questions, we performed a cross-sectional analysis based on baseline data from the Hakka Biobank (HKB) Prospective Study in Bobai, a county-based cohort study of Chinese adults aged ≥20 years. Participants were recruited by convenience sampling from 15 urban and rural grid sites in Bobai County between July and September 2024. Of 10,264 participants, 1,292 with incomplete questionnaire or laboratory data and 33 with malignancies or psychiatric disorders were excluded, leaving 8,939 for analysis (Figure 1). The Ethics Committee of Bobai County People’s Hospital approved the protocol (No. 2024-KY-026), and all participants provided written informed consent. As this was an observational, non-interventional study rather than a clinical trial, it was not registered on a clinical-trial platform, and no registration number is available.

### CKM staging and outcome

The CKM stages were defined according to the 2023 American Heart Association scientific statement and adapted to the Chinese population context (1, 17). Participants were categorized into five stages (0–4) based on metabolic risk factors, CKD status, and cardiovascular disease history. Stage 3 comprised subclinical CVD among participants with excess or dysfunctional adiposity, metabolic risk factors, or CKD, or a risk equivalent such as very-high-risk CKD or high predicted CVD risk. Subclinical CVD was assessed using the PREVENT risk equation. Stage 4 comprised clinically diagnosed coronary heart disease, heart failure, stroke, peripheral artery disease, or atrial fibrillation in the presence of CKM risk factors, based on self-reported history, with kidney failure considered in subclassification. Advanced CKM was defined as stages 3–4 and non-advanced CKM as stages 0–2. CKD was identified from estimated glomerular filtration rate calculated using the 2021 CKD-EPI equation and urine albumin-to-creatinine ratio according to KDIGO guidance (1, 18).

### Exposure, laboratory, and covariate assessment

After an overnight fast, blood and urine samples were collected under standardized conditions and analyzed centrally. Measurements included fasting glucose, triglycerides, total cholesterol, LDL-C, HDL-C, creatinine, blood urea nitrogen, serum uric acid (SUA), urinary albumin, and urinary creatinine. TyG index was calculated as ln[triglycerides (mg/dL) × fasting glucose (mg/dL)/2]. SUA was measured enzymatically. Each biomarker was assessed once at baseline, without repeat-sample reliability assessment.

The SUA was analyzed continuously per 50 μmol/L, per standard deviation, and using sex-specific hyperuricemia (HUA) thresholds of >420 μmol/L in men and >360 μmol/L in women (19). TyG was analyzed continuously, per standard deviation, by quartile, and dichotomously using 8.89, an internally derived ROC threshold without external validation.

Hypertension, diabetes, dyslipidemia, and obesity were defined according to Chinese clinical guidelines. Hypertension, diabetes, and dyslipidemia were identified when at least one disease-specific criterion was met: a measured value above the prespecified threshold, self-reported physician diagnosis, or current use of the corresponding medication. HUA was defined by the sex-specific measured threshold or self-reported history. Urate-lowering medication data were unavailable.

Covariates were prespecified from epidemiological evidence, clinical relevance, the CKM framework, and presumed temporal ordering, rather than automated selection. Model 2 included age, sex, ethnicity, education, marital status, smoking, alcohol use, physical activity, daytime napping, and nighttime sleep duration. Physical activity was classified as active for at least 150 min/week of moderate activity or 75 min/week of vigorous activity, and inactive otherwise. Standardized face-to-face interviews used a structured questionnaire previously applied in this cohort (19). Dietary purine and fructose intake, family history, medication adherence, and urate-lowering therapy were not collected.

### Statistical analysis

Baseline characteristics were compared by TyG category and HUA status. Continuous variables were summarized as mean ± standard deviation or median (interquartile range), as appropriate, and compared using Student’s *t*-test or the Mann–Whitney *U* test. Categorical variables were presented as frequencies and percentages and compared using the chi-square test. ROC analysis with the maximum Youden index identified the TyG threshold of 8.89 for advanced CKM. Because the threshold and performance estimates were derived in the same dataset without independent validation, they were considered exploratory and potentially optimistic.

Associations of TyG and SUA with advanced CKM were estimated using logistic regression. Model 1 was unadjusted; Model 2 included all prespecified covariates simultaneously using the enter method. No univariable screening or stepwise selection was used. Hypertension, diabetes, dyslipidemia, metabolic syndrome, and related medication exposures were excluded from primary adjustment because these conditions contribute to CKM staging and medication use contributes to their operational definitions. Re-entering them could duplicate outcome-defining information and introduce incorporation bias, collinearity, or overadjustment. Antihypertensive, glucose-lowering, and lipid-lowering medications were examined only in sensitivity analyses.

TyG was modeled per unit, per standard deviation, by quartile, and using the 8.89 cut-off; SUA was modeled per 50 μmol/L, per standard deviation, and by HUA status. Results are reported as odds ratios with 95% confidence intervals. Multicollinearity was assessed using variance inflation factors (VIFs) for continuous and binary predictors and adjusted generalized VIFs [GVIF^^(1/(2 × df))^] for multicategory variables. A value ≥5 was prespecified as concerning. All values were <2.0 (Supplementary Table S6). Nighttime sleep and daytime napping were retained because they represent distinct rest-related behaviors. The primary exposure model included 14 independent parameters and 1,164 advanced CKM events, yielding approximately 83.1 events per parameter and supporting model stability (20).

Restricted cubic splines tested global and nonlinear associations of TyG and SUA with advanced CKM. Sex-stratified splines and exposure-by-sex interactions assessed heterogeneity. Joint analyses classified participants as low TyG/non-HUA, high TyG/non-HUA, low TyG/HUA, or high TyG/HUA, with low TyG/non-HUA as the reference. Prespecified subgroup analyses were conducted by sex, age, ethnicity, education, and marital status, with interaction testing where appropriate. These parameter-intensive analyses were considered exploratory.

All sensitivity analyses yielded consistent association patterns with primary results, verifying the reliability of the core findings.

Incremental discrimination was assessed by comparing the covariate-only model with a model jointly adding TyG and SUA. The area under the ROC curve, DeLong test, and continuous net reclassification improvement were calculated. These measures represent internal, in-sample performance rather than prospective predictive or clinical utility; external validation and calibration are required.

All statistical analyses were conducted in R (version 4.4.2) and SPSS (version 23.0; IBM Corp., Armonk, NY, United States). A two-sided *p* < 0.05 was considered statistically significant.

## Results

### Primary demographics of the study population

The analysis included a total of 8,939 participants, of whom 65.63% were women, and 1,164 participants with advanced CKM. Han participants accounted for 99.06%. TyG 8.89 was the same-dataset ROC-derived threshold (Supplementary Table S1) and should not be interpreted as a validated clinical cut-off. Participants with TyG > 8.89 or HUA had a less favorable cardiometabolic profile (Supplementary Tables S2, S3). Overall, those with advanced CKM were older and had higher BMI, blood pressure, fasting glucose, triglycerides, TyG index, SUA, and UACR, besides lower HDL-C and eGFR (all *p* < 0.001; Table 1). Advanced CKM was also more prevalent in women and in participants with lower educational level, smoking, alcohol drinking, hypertension, diabetes, hyperlipidemia, HUA, and related medication use, whereas ethnicity, daytime napping, and Hakka status were comparable between groups.

**Table 1:** Baseline characteristics of participants according to advanced CKM syndrome status.

Variables	Total (n = 8,939)	Non advance CKM (n = 7,775)	advance CKM (n = 1,164)	*P*
Age, mean ± SD	55.38 ± 10.54	53.82 ± 9.45	65.80 ± 11.48	<0.001
BMI, mean ± SD	24.61 ± 3.36	24.56 ± 3.33	24.94 ± 3.50	<0.001
Nighttime sleep duration, mean (SD)	7.72 ± 1.73	7.69 ± 1.78	7.91 ± 1.40	<0.001
SBP, mean ± SD	133.85 ± 21.55	130.38 ± 18.74	157.05 ± 24.55	<0.001
DBP, mean ± SD	85.54 ± 12.05	84.16 ± 10.77	94.77 ± 15.57	<0.001
FBG, mean ± SD	5.46 ± 1.50	5.36 ± 1.36	6.12 ± 2.10	<0.001
TG, M (Q₁, Q₃)	1.48 (1.04, 2.18)	1.46 (1.03, 2.14)	1.62 (1.12, 2.42)	<0.001
TC, mean ± SD	5.15 ± 1.03	5.17 ± 0.99	5.02 ± 1.28	<0.001
HDL C, mean ± SD	1.25 ± 0.27	1.26 ± 0.27	1.18 ± 0.26	<0.001
LDL C, mean ± SD	3.15 ± 0.78	3.16 ± 0.77	3.05 ± 0.89	<0.001
TyG, mean ± SD	8.81 ± 0.65	8.78 ± 0.64	9.00 ± 0.73	<0.001
SUA, mean ± SD	346.80 ± 88.96	342.39 ± 87.09	376.25 ± 95.50	<0.001
eGFR, mean ± SD	101.28 ± 14.71	103.53 ± 12.14	86.25 ± 20.46	<0.001
UACR, M (Q₁, Q₃)	9.19 (5.48, 15.65)	8.57 (5.22, 13.61)	19.23 (9.99, 56.69)	<0.001
Sex, *n*(%)				<0.001
Man	3,072 (34.37)	2,502 (32.18)	570 (48.97)	
Woman	5,867 (65.63)	5,273 (67.82)	594 (51.03)	
Education level, *n*(%)				<0.001
Less than Primary school	5,601 (62.66)	4,800 (61.74)	801 (68.81)	
Middle/High school or equivalent	1833 (20.51)	1,594 (20.50)	239 (20.53)	
College or above	1,505 (16.84)	1,381 (17.76)	124 (10.65)	
Ethnicity, n(%)				0.502
Han	8,855 (99.06)	7,704 (99.09)	1,151 (98.88)	
Other	84 (0.94)	71 (0.91)	13 (1.12)	
Marital Status, *n*(%)				<0.001
Married	8,455 (94.59)	7,391 (95.06)	1,064 (91.41)	
Other	484 (5.41)	384 (4.94)	100 (8.59)	
Smoking status, *n*(%)				<0.001
Never	7,723 (86.40)	6,773 (87.11)	950 (81.62)	
Former	183 (2.05)	137 (1.76)	46 (3.95)	
Current	1,033 (11.56)	865 (11.13)	168 (14.43)	
Alcohol drink, *n*(%)				<0.001
Never	8,239 (92.17)	7,189 (92.46)	1,050 (90.21)	
Former	85 (0.95)	59 (0.76)	26 (2.23)	
Current	615 (6.88)	527 (6.78)	88 (7.56)	
Physical activity, *n*(%)				0.001
Inactive	4,204 (47.03)	3,707 (47.68)	497 (42.70)	
Active	4,735 (52.97)	4,068 (52.32)	667 (57.30)	
Daytime napping, *n*(%)				0.121
Never	1,473 (16.48)	1,278 (16.44)	195 (16.75)	
Occasional	2053 (22.97)	1813 (23.32)	240 (20.62)	
Often	5,413 (60.55)	4,684 (60.24)	729 (62.63)	
Hypertension, *n*(%)				<0.001
No	4,415 (49.39)	4,288 (55.15)	127 (10.91)	
Yes	4,524 (50.61)	3,487 (44.85)	1,037 (89.09)	
Antihypertensive Drug, *n*(%)				<0.001
No	7,561 (84.58)	6,943 (89.30)	618 (53.09)	
Yes	1,378 (15.42)	832 (10.70)	546 (46.91)	
Diabetes, *n*(%)				<0.001
No	7,987 (89.35)	7,181 (92.36)	806 (69.24)	
Yes	952 (10.65)	594 (7.64)	358 (30.76)	
Hypoglycemic drugs, *n*(%)				<0.001
No	8,475 (94.81)	7,520 (96.72)	955 (82.04)	
Yes	464 (5.19)	255 (3.28)	209 (17.96)	
Hyperlipidemia, *n*(%)				0.003
No	2,822 (31.57)	2,499 (32.14)	323 (27.75)	
Yes	6,117 (68.43)	5,276 (67.86)	841 (72.25)	
Lipid Lowering Drugs, *n*(%)				<0.001
No	8,643 (96.69)	7,581 (97.50)	1,062 (91.24)	
Yes	296 (3.31)	194 (2.50)	102 (8.76)	
Hakka, *n*(%)				0.321
No	6,966 (77.93)	6,072 (78.10)	894 (76.80)	
Yes	1973 (22.07)	1703 (21.90)	270 (23.20)	
TyG group, *n*(%)				<0.001
≤8.89	4,469 (49.99)	3,999 (51.43)	470 (40.38)	
>8.89	4,470 (50.01)	3,776 (48.57)	694 (59.62)	
Hyperuricemia, *n*(%)				<0.001
Non-HUA	6,162 (68.93)	5,498 (70.71)	664 (57.04)	
HUA	2,777 (31.07)	2,277 (29.29)	500 (42.96)	

TyG index, triglyceride-glucose index; SD, standard deviation; BMI, body mass index; SBP, systolic blood pressure; DBP, diastolic blood pressure; FPG, fasting plasma glucose; TG, triglyceride; TC, total cholesterol; LDL_C, low-density lipoprotein cholesterol; HDL_C, high-density lipoprotein cholesterol; SUA, serum uric acid; eGFR, estimated glomerular filtration rate, HUA, hyperuricemia; UACR, urine albumin-to-creatinine ratio; CKM, cardiovascular-kidney-metabolic.

### Independent associations of TyG index and SUA with CKM

In both univariable and multivariable logistic regression analyses, higher TyG index and SUA were associated with greater odds of advanced CKM (Table 2). After full multivariable adjustment, each 1-unit increment in TyG corresponded to 81% higher odds of advanced CKM (OR 1.81, 95% CI 1.64–2.01), and each-standard-deviation rise in TyG was associated with 48% higher odds (OR 1.48, 95% CI 1.38–1.58). Participants with TyG > 8.89 exhibited significantly elevated odds of advanced CKM relative to those with TyG ≤ 8.89 (OR 1.95, 95% CI 1.69–2.24).

For SUA, per-50 μmol/L increment was associated with 20% higher odds of advanced CKM following full adjustment (OR 1.20, 95% CI 1.15–1.25). Each-standard-deviation increase in SUA conferred 38% higher odds (OR 1.38, 95% CI 1.28–1.48), and individuals with HUA showed 86% higher odds compared with non-HUA counterparts (OR 1.86, 95% CI 1.62–2.15). Quartile-based analyses produced consistent results: relative to the lowest quartile, the highest quartile of TyG and SUA was linked to substantially higher odds of advanced CKM (TyG: OR 2.61, 95% CI 2.13–3.21; SUA: OR 2.03, 95% CI 1.64–2.50), with significant dose–response trends for both markers (all *P* for trend <0.001) (Table 2).

**Table 2:** Multivariate-adjusted logistic regression models of TyG index and SUA for advanced CKM syndrome.

Variables	Model1		Model2	
	OR (95%CI)	*P*	OR (95%CI)	*P*
TyG				
Per unit	1.59 (1.45 ~ 1.73)	<0.001	1.81 (1.64 ~ 2.01)	<0.001
Per SD	1.35 (1.28 ~ 1.43)	<0.001	1.48 (1.38 ~ 1.58)	<0.001
≤8.89	1.00 (Reference)		1.00 (Reference)	
>8.89	1.81 (1.60 ~ 2.04)	<0.001	1.95 (1.69 ~ 2.24)	<0.001
TyG quantile				
Q1	1.00 (Reference)		1.00 (Reference)	
Q2	1.31 (1.08 ~ 1.58)	0.006	1.36 (1.09 ~ 1.69)	0.006
Q3	1.40 (1.16 ~ 1.69)	<0.001	1.37 (1.10 ~ 1.70)	0.004
Q4	2.23 (1.87 ~ 2.67)	<0.001	2.61 (2.13 ~ 3.21)	<0.001
Ptrend		<0.001		<0.001
SUA				
Per 50 unit	1.22 (1.18 ~ 1.26)	<0.001	1.20 (1.15 ~ 1.25)	<0.001
Per SD	1.43 (1.35 ~ 1.52)	<0.001	1.38 (1.28 ~ 1.48)	<0.001
Non-HUA	1.00 (Reference)		1.00 (Reference)	
HUA	1.82 (1.60 ~ 2.06)	<0.001	1.86 (1.62 ~ 2.15)	<0.001
SUA quantile				
Q1	1.00 (Reference)		1.00 (Reference)	
Q2	1.03 (0.84 ~ 1.26)	0.777	0.91 (0.72 ~ 1.14)	0.387
Q3	1.62 (1.35 ~ 1.96)	<0.001	1.30 (1.05 ~ 1.61)	0.015
Q4	2.49 (2.08 ~ 2.97)	<0.001	2.03 (1.64 ~ 2.50)	<0.001
Ptrend		<0.001		<0.001

Model 1 was unadjusted. Model 2 was adjusted for age, sex, ethnicity, educational level, marital status, smoking status, alcohol consumption, physical activity, nighttime sleep duration, and daytime napping. All covariates were prespecified and entered simultaneously using the enter method. All variance inflation factors were <2.0. TyG index, triglyceride-glucose index; SUA, serum uric acid; HUA, hyperuricemia; HUA, hyperuricemia; OR, odds ratio; CI, confidence interval.

### Dose–response relationships of TyG and SUA with advanced CKM

**Figure 2:**
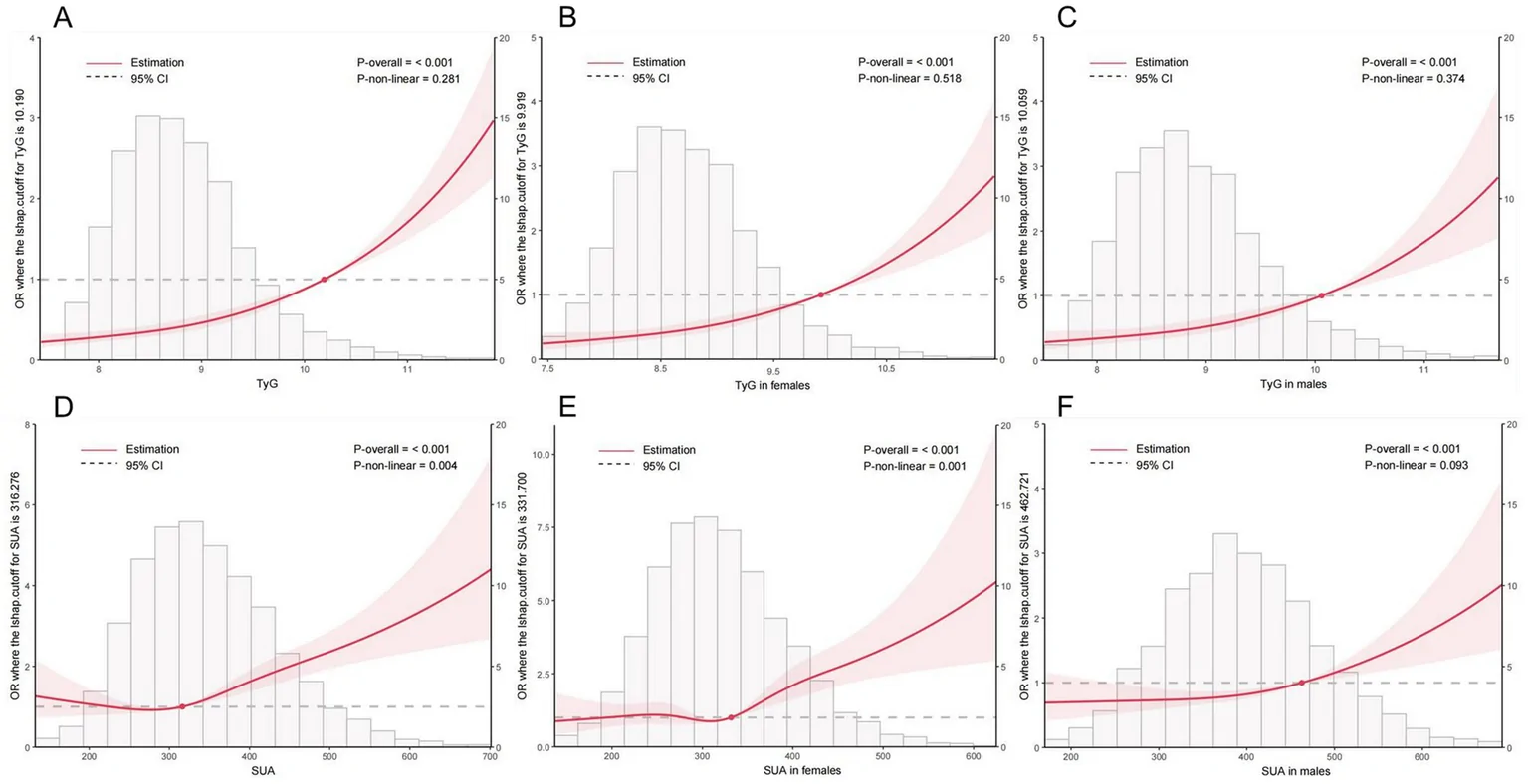
Restricted cubic spline analyses of the associations of the TyG index and serum uric acid with advanced CKM syndrome in the overall population and by sex. Panels **(A–C)** show the dose–response association between the TyG index and advanced cardiovascular-kidney-metabolic (CKM) syndrome in the overall population, women, and men, respectively. Panels **(D–F)** show the corresponding associations for serum uric acid (SUA). Models were adjusted for age, sex, ethnicity, educational level, marital status, smoking status, alcohol drinking, physical activity, daytime napping, and nighttime sleep duration. Solid lines indicate adjusted odds ratios, and shaded areas indicate 95% confidence intervals. Histograms represent the distribution of the exposure variables.

The RCS analyses revealed distinct exposure-response patterns for TyG and SUA (Figure 2). In the overall population, TyG was significantly associated with advanced CKM (*P*-overall < 0.001), with no evidence of non-linearity, indicating an approximately linear positive association. Sex-stratified spline analyses showed similar patterns in both women and men, with significant overall associations but no significant nonlinear component.

By comparison, SUA exhibited a statistically significant non-linearity in its relationship with advanced CKM in the full cohort (overall association *p* < 0.001; non-linearity *p* = 0.004), reflecting a J-shaped pattern. When analyses were stratified by sex, this curvilinear trend was more pronounced among women (overall *p* < 0.001; non-linear *p* = 0.001). In men, although the general association remained robust (overall *p* < 0.001), evidence for a nonlinear component was not statistically supported (non-linear *p* = 0.093).

### Joint associations of TyG index and SUA with advanced CKM

When participants were categorized jointly by TyG and HUA status, those with both high TyG and HUA had the highest odds of advanced CKM. Compared with the low TyG/non-HUA group, the high TyG/HUA group had an unadjusted OR of 2.63 (95% CI 2.23–3.11; *p* < 0.001), which remained robust after multivariable adjustment (adjusted OR 3.01, 95% CI 2.49–3.64) (Table 3). Further analysis demonstrated that those with advanced CKM were at significantly greater risk regardless of TyG and SUA levels (Table 4).

**Table 3:** Risk of advanced CKM syndrome upon co-exposure stratified by TyG index and SUA.

Variables	Model1		Model2	
	OR (95%CI)	*P*	OR (95%CI)	*P*
TyG ≤ 8.89 and non-HUA	1.00 (Reference)		1.00 (Reference)	
TyG > 8.89 and non-HUA	1.85 (1.58 ~ 2.18)	<0.001	1.86 (1.55 ~ 2.23)	<0.001
TyG ≤ 8.89 and HUA	1.94 (1.61 ~ 2.34)	<0.001	1.78 (1.44 ~ 2.20)	<0.001
TyG > 8.89 and HUA	2.63 (2.23 ~ 3.11)	<0.001	3.01 (2.49 ~ 3.64)	<0.001

Model 1 was Crude. Model 2 was adjusted for age, sex, ethnicity, educational level, marital status, smoking status, alcohol drinking, physical activity, daytime napping, and nighttime sleep duration. TyG index, triglyceride-glucose index; SUA, serum uric acid; HUA, hyperuricemia; HUA, hyperuricemia; OR, odds ratio; CI, confidence interval.

**Table 4:** Risk reclassification of advanced CKM syndrome based on TyG index and SUA.

Variables	Model1		Model2	
	OR (95%CI)	*P*	OR (95%CI)	*P*
HUA				
TyG ≤ 8.89	1.00 (Reference)		1.00 (Reference)	
TyG > 8.89	1.36 (1.11 ~ 1.66)	0.002	1.68 (1.34 ~ 2.11)	<0.001
Non-HUA				
TyG ≤ 8.89	1.00 (Reference)		1.00 (Reference)	
TyG > 8.89	1.85 (1.585 ~ 2.18)	<0.001	1.86 (1.55 ~ 2.24)	<0.001
TyG ≤ 8.89				
Non-HUA	1.00 (Reference)		1.00 (Reference)	
HUA	1.94 (1.61 ~ 2.34)	<0.001	1.77 (1.43 ~ 2.19)	<0.001
TyG > 8.89				
Non-HUA	1.00 (Reference)		1.00 (Reference)	
HUA	1.42 (1.19 ~ 1.69)	<0.001	1.62 (1.33 ~ 1.98)	<0.001

Model 1 was Crude. Model 2 was adjusted for age, sex, ethnicity, educational level, marital status, smoking status, alcohol drinking, physical activity, daytime napping, and nighttime sleep duration. TyG index, triglyceride-glucose index; SUA, serum uric acid; HUA, hyperuricemia; HUA, hyperuricemia; OR, odds ratio; CI, confidence interval.

### Sensitivity analyses

Sensitivity analyses confirmed the robustness of our main findings. Additional sensitivity analyses (Supplementary Table S4), including medication adjustment, exclusion of participants with renal impairment and those receiving metabolic drugs, yielded consistent results across six analytical scenarios. The group with both elevated TyG index and HUA consistently showed the greatest risk of advanced CKM, with no substantial attenuation of odds ratios. In female-only analyses (Supplementary Table S5), independent associations of TyG index and SUA with advanced CKM persisted, and further adjustment for menopausal status produced minimal changes in effect sizes. Women with concurrent elevated TyG index and HUA had the highest odds of advanced CKM (Model 3: OR = 3.814, 95%CI 2.957–4.920, *p* < 0.001), with a risk pattern comparable to the overall cohort.

### Apparent incremental discrimination and reclassification

Within the same dataset, adding TyG and SUA to the conventional risk model significantly improved discrimination for advanced CKM (Table 5), increasing the AUC from 0.797 to 0.816 (ΔAUC = 0.019, DeLong test *p* < 0.001). Continuous NRI analysis further demonstrated a substantial improvement in risk reclassification (NRI = 0.433, 95% CI 0.372–0.496), including an NRI+ of 0.125 (95% CI 0.070–0.186) and an NRI− of 0.308 (95% CI 0.287–0.329).

**Table 5:** Incremental predictive value of TyG and SUA for advanced CKM.

Outcome	Estimate	95%CI
AUC of base model	0.7973	–
AUC of base + TyG + SUA model	0.8160	–
ΔAUC	0.0187	0.0136 to 0.0239
DeLong test *Z*	−7.118	–
DeLong test *p*-value	1.095e-12	–
Continuous NRI	0.4332	0.3721 to 0.4959
NRI+	0.1254	0.0704 to 0.1864
NRI−	0.3078	0.2869 to 0.3289

### Stratified analysis of demographic characteristics

**Figure 3:**
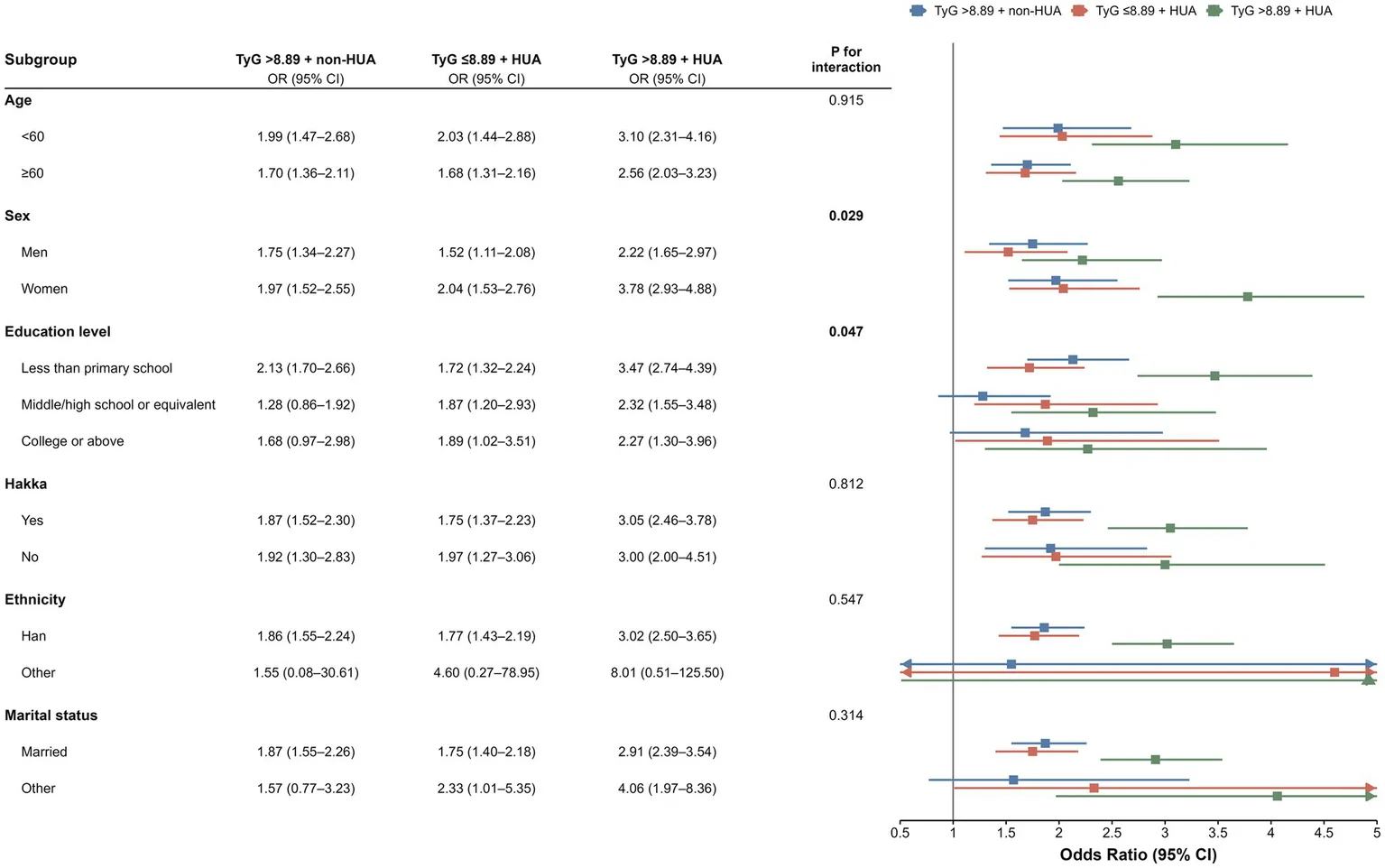
Stratified and interaction analyses for associations of TyG index and SUA with advanced CKM syndrome. Results are presented across pre-specified subgroups. TyG ≤ 8.89 plus non-HUA served as the reference group. Blue squares: TyG > 8.89 + non-HUA; red squares: TyG ≤ 8.89 + HUA; green squares: TyG > 8.89 + HUA. Horizontal bars represent 95% confidence intervals. P-for-interaction values test statistical interaction between combined TyG-HUA status and each subgroup factor. Stratified analyses were performed based on model2, wherein the models were not adjusted for the variables used for stratification themselves. TyG index, triglyceride-glucose index; SUA, serum uric acid; HUA, hyperuricemia; HUA, hyperuricemia; OR: Odds Ratio, CI: Confidence Interval.

Subgroup analyses showed that the association of the combined high-TyG/HUA phenotype with advanced CKM was generally consistent across age, sex, ethnicity, marital status, and education (Figure 3). Notably, the association was significantly stronger in women (*P* for interaction = 0.028) and was also significantly associated in participants with less than primary school education (*p* = 0.047 for interaction).

## Discussion

In this community-focused study of Chinese adults, higher TyG index and SUA were associated with advanced CKM, but their association patterns were not identical. TyG showed a largely linear association across its distribution, whereas SUA exhibited a nonlinear J-shaped relationship. Individuals with concomitantly elevated TyG and HUA had the greatest observed odds of advanced CKM, and the addition of both markers improved discrimination and risk reclassification beyond conventional factors. These findings suggest that TyG and SUA reflect distinct yet complementary dimensions of CKM vulnerability and may collectively improve identification of individuals with a high probability of existing advanced disease.

The approximately linear association observed for TyG is biologically plausible given its close relationship with insulin resistance, an important feature of CKM pathophysiology. Insulin resistance is associated with dysglycemia, dyslipidemia, adipose tissue dysfunction, endothelial injury, low-grade inflammation, and altered kidney hemodynamics, thereby linking metabolic abnormalities with cardiovascular and kidney dysfunction (21–23). As a composite index derived from fasting triglyceride and glucose concentrations, TyG may capture a graded burden of insulin resistance-related metabolic dysfunction across the CKM continuum. Our findings are consistent with previous observational studies linking higher TyG levels to diabetes, hypertension, atherosclerosis, CKD, and adverse cardiovascular outcomes (7, 24), and extend this evidence by situating TyG within the integrated CKM framework.

Nevertheless, TyG is a surrogate rather than a direct measure of insulin resistance. The hyperinsulinemic-euglycemic clamp remains the reference method, whereas HOMA-IR requires fasting insulin (4, 5). TyG is more feasible in population settings because it uses routinely measured fasting triglycerides and glucose (11). However, fasting insulin and clamp measurements were unavailable; therefore, we could not compare TyG directly with HOMA-IR or the clamp or establish its superiority. Moreover, because TyG and SUA were added jointly to the conventional model, the observed improvement in in-sample discrimination and reclassification cannot be attributed to TyG alone. Prospective studies incorporating established insulin-resistance measures and external validation are needed to determine the incremental clinical value of TyG.

In contrast, the J-shaped association of SUA suggests a more complex biological role. Unlike TyG, which primarily reflects insulin resistance-related metabolic burden, SUA may capture the combined influence of urate production, renal excretion, oxidative stress, endothelial dysfunction, vascular inflammation, and altered tubular handling (25–27). At higher concentrations, uric acid may interact with these metabolic disturbances via shared oxidative stress pathways, impair nitric oxide bioavailability, and has been linked to microvascular and renal injury, thereby amplifying CKM risks (25, 28–31). Conversely, associations at low SUA concentrations reported in previous U- or J-shaped studies may reflect poor nutritional status, chronic illness, medication effects, or reduced antioxidant capacity (32). Under this framework, SUA is better understood not as a purely linear risk factor, but as a threshold-sensitive marker of metabolic-vascular and renal stress (27). However, both extremes may be confounded, and the present cross-sectional spline cannot establish a causal optimum or treatment threshold. These findings further suggest that TyG and SUA reflect distinct, rather than interchangeable, aspects of CKM pathobiology.

A further important finding was the joint association of elevated TyG and SUA. Participants with both high TyG and HUA had the highest odds of advanced CKM, even after adjustment for conventional covariates. The joint association remained broadly consistent after additional medication adjustment and after excluding participants with renal impairment or metabolic medication use, although residual confounding by drug subclasses, dosage, duration, adherence, and urate-lowering therapy remains possible. This pattern is biologically plausible, as insulin resistance and urate dysregulation are closely interconnected and may potentially amplify one another via putative shared pathways involving oxidative stress, endothelial dysfunction, chronic inflammation, adiposity, and renal tubular reabsorption (15, 33, 34). Clinically, this combined phenotype may represent a readily identifiable state of convergent metabolic-renal-vascular stress. The incremental improvement in AUC and reclassification metrics showed apparent in-sample improvement, further suggesting that integrating TyG and SUA into risk assessment may provide information not fully captured by conventional factors alone.

We observed sex-related heterogeneity, with a stronger combined TyG–HUA association and a clearer nonlinear SUA pattern in women. Several mechanisms may contribute to this finding. Before menopause, estrogen-related physiology is associated with lower SUA levels and greater renal urate clearance, whereas declining estrogen during and after menopause may alter renal urate handling, increase SUA, and coincide with impaired insulin sensitivity and vascular dysfunction (35–37). The menopausal transition is also accompanied by a shift from gluteofemoral to abdominal and visceral adiposity, which may promote hypertriglyceridemia, insulin resistance, low-grade inflammation, and greater CKM burden (35, 38). Sex differences in endothelial function and oxidative balance, together with the use of sex-specific HUA thresholds, may further influence the observed association. Nevertheless, these explanations remain hypotheses, and the interaction findings should be interpreted cautiously because of the cross-sectional design, possible multiple testing, and residual confounding by hormone therapy, reproductive history, and directly measured body-fat distribution. Thus, the TyG–SUA profile may be particularly relevant for characterizing CKM burden in women, but prospective studies stratified by menopausal status are required before drawing sex-specific clinical conclusions.

By contrast, the stronger association among participants with lower educational attainment may reflect social rather than biological differences, because lower education may be associated with poorer diet, lower health literacy, delayed healthcare access, and less effective long-term risk-factor management. This finding should therefore be interpreted within a social-vulnerability framework and externally validated before informing targeted interventions.

These findings suggest that TyG and SUA may capture complementary metabolic and renal vulnerabilities and could serve as practical adjuncts to CKM assessment in community settings. TyG can be calculated from routine fasting glucose and triglyceride measurements, while SUA is available. Elevated or discordant values may prompt repeat testing under stable conditions and evaluation of blood pressure, glycemia, lipids, eGFR, UACR, cardiovascular history, lifestyle, and medications. However, the internally derived TyG threshold of 8.89 should not be used as a screening rule, and neither biomarker should independently trigger treatment. Prospective validation is required to define algorithms, thresholds, and follow-up strategies.

Several limitations warrant consideration. First, the cross-sectional design precludes temporal or causal inference and remains susceptible to reverse causation. Second, single TyG and SUA measurements may be influenced by acute glycemia, diet, hydration, kidney function, and medications, potentially causing exposure misclassification. Third, residual confounding and self-report error remain possible because detailed dietary, family-history, medication, hormonal, and body-composition data were unavailable, although menopausal-status adjustment minimally changed estimates among women. Fourth, recruitment from one county in Guangxi, with predominantly Han Chinese and female participants, limits generalizability. Fifth, the internally derived TyG threshold of 8.89 lacks external validation, while the in-sample improvement after jointly adding TyG and SUA may be optimistic, cannot be attributed to TyG alone, and does not establish clinical utility. Sixth, unavailable fasting insulin and clamp measurements precluded comparison with HOMA-IR or direct insulin-sensitivity measures. Finally, although multicollinearity and overfitting were not evident in the primary model, spline, interaction, and subgroup analyses remain exploratory. Prospective multicenter studies with repeated measurements and external validation are required.

## Conclusion

In conclusion, higher TyG and SUA were cross-sectionally associated with advanced CKM, and their co-occurrence identified a subgroup with higher observed odds. These results support further evaluation of routine metabolic biomarkers as adjuncts to comprehensive CKM assessment, not as evidence of causation, treatment targets, or a validated screening algorithm. Prospective studies with repeated measurements, external validation, and representation across sex, menopausal status, ethnicity, and region are needed.

## Data Availability

The datasets generated and/or analyzed during the current study are not publicly available due to participant privacy and ethical restrictions but are available from the corresponding author on reasonable request. Requests to access the datasets should be directed to Zengnan Mo, mozengnan@gxmu.edu.cn.
