# Supplementary material for: Insulin resistance and uric acid-related metabolic disturbances jointly identify individuals at high risk of advanced cardiovascular-kidney-metabolic syndrome: a population-based study
Source: Front Nutr. 2026 Sep 3;13:1933816. doi: 10.3389/fnut.2026.1933816 (PMC13582521; doi:10.3389/fnut.2026.1933816)
Supplement: Supplementary file 1 [file Table_1.docx]

**Table S1 ROC curve analysis determined optimal cut-off value of the TyG index for predicting advanced CKM**

| Test | optimal cut-off value | Sensitivity | Specificity | AUC |
| --- | --- | --- | --- | --- |
| TyG index | 8.89 | 0.617 | 0.528 | 0.590 |

ROC curve, receiver operating characteristic curve; AUC, area under the curve; TyG index, triglyceride-glucose index.

**TableS2 Baseline characteristics of the study population according to TyG index**

| Variables | Total (n = 8939) | TyG≤8.89  (n = 5347) | TyG＞8.89  (n = 3592) | *P* |
| --- | --- | --- | --- | --- |
|  |  |  |  |  |
| Age, Mean (SD) | 55.38 (10.54) | 55.00 (10.71) | 55.95 (10.26) | <0.001 |
| BMI, Mean (SD) | 24.61 (3.36) | 24.02 (3.28) | 25.48 (3.28) | <0.001 |
| Sleep duration, Mean (SD) | 7.72 (1.73) | 7.69 (1.71) | 7.75 (1.76) | 0.140 |
| SBP, Mean (SD) | 133.85 (21.55) | 131.31 (21.42) | 137.64 (21.18) | <0.001 |
| DBP, Mean (SD) | 85.54 (12.05) | 83.85 (11.94) | 88.06 (11.78) | <0.001 |
| FPG, Mean (SD) | 5.46 (1.50) | 5.06 (0.64) | 6.06 (2.10) | <0.001 |
| TG, Mean (SD) | 1.93 (1.80) | 1.15 (0.34) | 3.09 (2.37) | <0.001 |
| TG, M (Q₁, Q₃) | 1.48 (1.04, 2.18) | 1.13 (0.89, 1.40) | 2.43 (1.98, 3.29) | <.00 |
| TC, Mean (SD) | 5.15 (1.03) | 4.99 (0.94) | 5.40 (1.12) | <0.001 |
| HDL_C, Mean (SD) | 1.25 (0.27) | 1.34 (0.26) | 1.11 (0.22) | <0.001 |
| LDL_C, Mean (SD) | 3.15 (0.78) | 3.04 (0.76) | 3.30 (0.80) | <0.001 |
| SUA, Mean (SD) | 346.80 (88.96) | 328.62 (82.35) | 373.85 (91.52) | <0.001 |
| eGFR, Mean (SD) | 101.28 (14.71 | 101.96 (14.13) | 100.27 (15.48) | <0.001 |
| UACR, M (Q₁, Q₃) | 9.19 (5.48, 15.65) | 8.57 (5.13, 13.70) | 10.43 (6.10, 19.71) | <0.001 |
| Night Shift, n(%) |  |  |  | 0.501 |
| 1 | 2269 (29.95) | 1385 (30.24) | 884 (29.52) |  |
| 2 | 5306 (70.05) | 3195 (69.76) | 2111 (70.48) |  |
| CKM stage, n(%) |  |  |  | <0.001 |
| stage 0 | 999 (11.18) | 995 (18.61) | 4 (0.11) |  |
| stage 1 | 1330 (14.88) | 1303 (24.37) | 27 (0.75) |  |
| stage 2 | 5446 (60.92) | 2500 (46.76) | 2946 (82.02) |  |
| stage 3 | 908 (10.16) | 411 (7.69) | 497 (13.84) |  |
| stage 4 | 256 (2.86) | 138 (2.58) | 118 (3.29) |  |
| CKM advance, n(%) |  |  |  | <0.001 |
| Non advance | 7775 (86.98) | 4798 (89.73) | 2977 (82.88) |  |
| Advance | 1164 (13.02) | 549 (10.27) | 615 (17.12) |  |
| Hyperuricemia, n(%) |  |  |  | <0.001 |
| Non-HUA | 6162 (68.93) | 4105 (76.77) | 2057 (57.27) |  |
| HUA | 2777 (31.07) | 1242 (23.23) | 1535 (42.73) |  |
| Sex, n(%) |  |  |  | <0.001 |
| Man | 3072 (34.37) | 1659 (31.03) | 1413 (39.34) |  |
| Woman | 5867 (65.63) | 3688 (68.97) | 2179 (60.66) |  |
| Education level, n(%) |  |  |  | 0.003 |
| Less than Primary school | 5601 (62.66) | 3415 (63.87) | 2186 (60.86) |  |
| Middle/High school or equivalent | 1833 (20.51) | 1035 (19.36) | 798 (22.22) |  |
| College or above | 1505 (16.84) | 897 (16.78) | 608 (16.93) |  |
| Ethnicity, n(%) |  |  |  | 0.016 |
| Han | 8855 (99.06) | 5286 (98.86) | 3569 (99.36) |  |
| Other | 84 (0.94) | 61 (1.14) | 23 (0.64) |  |
| Marital Status, n(%) |  |  |  |  |
| Married | 8455 (94.59) | 5051 (94.46) | 3404 (94.77) | 0.536 |
| Other | 484 (5.41) | 296 (5.54) | 188 (5.23) |  |
| Smoking status, n(%) |  |  |  | <0.001 |
| Never | 7723 (86.40) | 4751 (88.85) | 2972 (82.74) |  |
| Former | 183 (2.05) | 100 (1.87) | 83 (2.31) |  |
| Current | 1033 (11.56) | 496 (9.28) | 537 (14.95) |  |
| Alcohol drink, n(%) |  |  |  | <0.001 |
| Never | 8239 (92.17) | 5031 (94.09) | 3208 (89.31) |  |
| Former | 85 (0.95) | 52 (0.97) | 33 (0.92) |  |
| Current | 615 (6.88) | 264 (4.94) | 351 (9.77) |  |
| Physical activity, n(%) |  |  |  | 0.007 |
| Inactive | 4204 (47.03) | 2577 (48.20) | 1627 (45.30) |  |
| Active | 4735 (52.97) | 2770 (51.80) | 1965 (54.70) |  |
| Daytime napping, n(%) |  |  |  | <0.001 |
| Never | 1473 (16.48) | 950 (17.77) | 523 (14.56) |  |
| Occasional | 2053 (22.97) | 1216 (22.74) | 837 (23.30) |  |
| Often | 5413 (60.55) | 3181 (59.49) | 2232 (62.14) |  |
| Hypertension, n(%) |  |  |  | <0.001 |
| No | 4415 (49.39) | 2948 (55.13) | 1467 (40.84) |  |
| Yes | 4524 (50.61) | 2399 (44.87) | 2125 (59.16) |  |
| Antihypertensive drugs, n(%) |  |  |  | <0.001 |
| No | 7561 (84.58) | 4683 (87.58) | 2878 (80.12) |  |
| Yes | 1378 (15.42) | 664 (12.42) | 714 (19.88) |  |
| Diabetes, n(%) |  |  |  | <0.001 |
| No | 7987 (89.35) | 5144 (96.20) | 2843 (79.15) |  |
| Yes | 952 (10.65) | 203 (3.80) | 749 (20.85) |  |
| Hypoglycemic drugs, n(%) |  |  |  | <0.001 |
| No | 8475 (94.81) | 5223 (97.68) | 3252 (90.53) |  |
| Yes | 464 (5.19) | 124 (2.32) | 340 (9.47) |  |
| Hyperlipidemia, n(%) |  |  |  | <0.001 |
| No | 2822 (31.57) | 2695 (50.40) | 127 (3.54) |  |
| Yes | 6117 (68.43) | 2652 (49.60) | 3465 (96.46) |  |
| Lipid Lowering Drugs, n(%) |  |  |  | <0.001 |
| No | 8643 (96.69) | 5227 (97.76) | 3416 (95.10) |  |
| Yes | 296 (3.31) | 120 (2.24) | 176 (4.90) |  |
| Hakka, n(%) |  |  |  | <0.001 |
| Yes | 6966 (77.93) | 4077 (76.25) | 2889 (80.43) |  |
| No | 1973 (22.07) | 1270 (23.75) | 703 (19.57) |  |

*TyG index* triglyceride-glucose index, *SD* standard deviation, *BMI* Body Mass Index, *SBP* Systolic Blood Pressure, *DBP* Diastolic Blood Pressure, *FPG* Fasting Plasma Glucose, *TG* Triglyceride, *TC* Total Cholesterol, *LDL_C* Low-Density Lipoprotein Cholesterol, *HDL_C* High-Density Lipoprotein Cholesterol, *SUA* serum uric acid, *eGFR* Estimated Glomerular Filtration Rate, *HUA* Hyperuricemia, *UACR* Urine Albumin-to-Creatinine Ratio, *CKM* cardiovascular-kidney-metabolic.

**TableS3 Baseline characteristics of the study population according to SUA**

| Variables | Total (n = 8939) | Non-HUA  (n = 6162) | HUA  (n = 2777) | *P* |
| --- | --- | --- | --- | --- |
|  |  |  |  |  |
| Age, Mean (SD) | 55.38 (10.54) | 55.10 (10.39) | 55.99 (10.84) | <0.001 |
| BMI, Mean (SD) | 24.61 ± 3.36 | 24.13 ± 3.25 | 25.67 ± 3.35 | <0.001 |
| Sleep duration, Mean (SD) | 7.72 (1.73) | 7.72 (1.74) | 7.71 (1.72) | 0.731 |
| SBP, Mean (SD) | 133.85 (21.55) | 132.32 (21.44) | 137.26 (21.40) | <0.001 |
| DBP, Mean (SD) | 85.54 (12.05) | 84.38 (11.83) | 88.11 (12.15) | <0.001 |
| FBG, Mean (SD) | 5.46 (1.50) | 5.43 (1.57) | 5.51 (1.33) | 0.020 |
| TG, M (Q₁, Q₃) | 1.48 (1.04, 2.18) | 1.34 (0.97, 1.95) | 1.82 (1.31, 2.68) | <0.001 |
| TC, Mean (SD) | 5.15 (1.03) | 5.10 (1.00) | 5.26 (1.09) | <0.001 |
| HDL_C, Mean (SD) | 1.25 (0.27) | 1.27 (0.27) | 1.19 (0.25) | <0.001 |
| LDL_C, Mean (SD) | 3.15 (0.78) | 3.10 (0.77) | 3.25 (0.80) | <0.001 |
| eGFR, Mean (SD) | 101.28 (14.71) | 103.53 (12.61) | 96.31 (17.54) | <0.001 |
| UACR, M (Q₁, Q₃) | 9.19 (5.48, 15.65) | 9.02 (5.48, 14.59) | 9.81 (5.57, 19.01) | <0.001 |
| TyG, Mean (SD) | 8.81 (0.65) | 8.71 (0.63) | 9.03 (0.64) | <0.001 |
| TyG_group, n(%) |  |  |  | <0.001 |
| ≤8.89 | 5347 (59.82) | 4105 (66.62) | 1242 (44.72) |  |
| >8.89 | 3592 (40.18) | 2057 (33.38) | 1535 (55.28) |  |
| CKM stage, n(%) |  |  |  | <0.001 |
| stage 0 | 999 (11.18) | 875 (14.20) | 124 (4.47) |  |
| stage 1 | 1330 (14.88) | 1029 (16.70) | 301 (10.84) |  |
| stage 2 | 5446 (60.92) | 3594 (58.33) | 1852 (66.69) |  |
| stage 3 | 908 (10.16) | 513 (8.33) | 395 (14.22) |  |
| stage 4 | 256 (2.86) | 151 (2.45) | 105 (3.78) |  |
| CKM advance, n(%) |  |  |  | <0.001 |
| Non advance | 7775 (86.98) | 5498 (89.22) | 2277 (81.99) |  |
| advance | 1164 (13.02) | 664 (10.78) | 500 (18.01) |  |
| Sex, n(%) |  |  |  | <0.001 |
| Man | 3072 (34.37) | 1993 (32.34) | 1079 (38.85) |  |
| Woman | 5867 (65.63) | 4169 (67.66) | 1698 (61.15) |  |
| Education level, n(%) |  |  |  | 0.004 |
| Less than Primary school | 5601 (62.66) | 3931 (63.79) | 1670 (60.14) |  |
| Middle/High school or equivalent | 1833 (20.51) | 1221 (19.81) | 612 (22.04) |  |
| College or above | 1505 (16.84) | 1010 (16.39) | 495 (17.82) |  |
| Ethnicity, n(%) |  |  |  | 0.102 |
| Han | 8855 (99.06) | 6111 (99.17) | 2744 (98.81) |  |
| Other | 84 (0.94) | 51 (0.83) | 33 (1.19) |  |
| Marital Status, n(%) |  |  |  | 0.283 |
| Married | 8455 (94.59) | 5839 (94.76) | 2616 (94.20) |  |
| Other | 484 (5.41) | 323 (5.24) | 161 (5.80) |  |
| Smoking status, n(%) |  |  |  | 0.004 |
| Never | 7723 (86.40) | 5369 (87.13) | 2354 (84.77) |  |
| Former | 183 (2.05) | 111 (1.80) | 72 (2.59) |  |
| Current | 1033 (11.56) | 682 (11.07) | 351 (12.64) |  |
| Alcohol drink, n(%) |  |  |  | <0.001 |
| Never | 8239 (92.17) | 5731 (93.01) | 2508 (90.31) |  |
| Former | 85 (0.95) | 57 (0.93) | 28 (1.01) |  |
| Current | 615 (6.88) | 374 (6.07) | 241 (8.68) |  |
| Physical activity, n(%) |  |  |  | <0.001 |
| Inactive | 4204 (47.03) | 3008 (48.82) | 1196 (43.07) |  |
| Active | 4735 (52.97) | 3154 (51.18) | 1581 (56.93) |  |
| Daytime napping, n(%) |  |  |  | 0.033 |
| Never | 1473 (16.48) | 1054 (17.10) | 419 (15.09) |  |
| Occasional | 2053 (22.97) | 1424 (23.11) | 629 (22.65) |  |
| Often | 5413 (60.55) | 3684 (59.79) | 1729 (62.26) |  |
| Hypertension, n(%) |  |  |  | <0.001 |
| No | 4415 (49.39) | 3324 (53.94) | 1091 (39.29) |  |
| Yes | 4524 (50.61) | 2838 (46.06) | 1686 (60.71) |  |
| Antihypertensive drugs, n(%) |  |  |  | <0.001 |
| No | 7561 (84.58) | 5377 (87.26) | 2184 (78.65) |  |
| Yes | 1378 (15.42) | 785 (12.74) | 593 (21.35) |  |
| Diabetes, n(%) |  |  |  | 0.006 |
| No | 7987 (89.35) | 5543 (89.95) | 2444 (88.01) |  |
| Yes | 952 (10.65) | 619 (10.05) | 333 (11.99) |  |
| Hypoglycemic drugs,, n(%) |  |  |  | 0.310 |
| No | 8475 (94.81) | 5852 (94.97) | 2623 (94.45) |  |
| Yes | 464 (5.19) | 310 (5.03) | 154 (5.55) |  |
| Hyperlipidemia, n(%) |  |  |  | <0.001 |
| No | 2822 (31.57) | 2233 (36.24) | 589 (21.21) |  |
| Yes | 6117 (68.43) | 3929 (63.76) | 2188 (78.79) |  |
| Lipid Lowering Drugs, n(%) |  |  |  | 0.040 |
| No | 8643 (96.69) | 5974 (96.95) | 2669 (96.11) |  |
| Yes | 296 (3.31) | 188 (3.05) | 108 (3.89) |  |

*TyG index* triglyceride-glucose index, *SD* standard deviation, *BMI* Body Mass Index, *SBP* Systolic Blood Pressure, *DBP* Diastolic Blood Pressure, *FPG* Fasting Plasma Glucose, *TG* Triglyceride, *TC* Total Cholesterol, *LDL_C* Low-Density Lipoprotein Cholesterol, *HDL_C* High-Density Lipoprotein Cholesterol, *SUA* serum uric acid, *eGFR* Estimated Glomerular Filtration Rate, *CKM* cardiovascular-kidney-metabolic, *UACR* Urine Albumin-to-Creatinine Ratio, *CKD* chronic kidney disease.

Sensitivity analyses were conducted to assess whether the main associations were altered by kidney function status and medication use (Table S4). Analysis 1 further incorporated antihypertensive, lipid‑lowering and hypoglycaemic medications into the multivariable model. In Analysis 2 and 3, participants with reduced eGFR or elevated UACR were sequentially excluded. Analyses 4‑6 excluded participants taking antihypertensive agents, lipid‑lowering/hypoglycaemic agents, or all three categories of medications, respectively.

In all six sensitivity analyses, per‑unit TyG index and per‑50‑μmol/L SUA remained significantly associated with higher odds of advanced CKM (all ****P*<0.001). For the combined TyG‑HUA categories, the group with concurrently elevated TyG index and HUA persistently demonstrated the greatest risk for advanced CKM across all sensitivity models. Effect estimates fluctuated within a moderate range without material attenuation, suggesting that the observed associations were not driven by baseline kidney impairment or metabolic medication exposure.

**Table S4 Sensitivity analysis for the joint association of the TyG index and SUA with advanced CKM**

|  | OR (95% CI) | | | | | |
| --- | --- | --- | --- | --- | --- | --- |
|  | Analysis 1 | Analysis 2 | Analysis 3 | Analysis 4 | Analysis 5 | Analysis 6 |
| TyG Per unit | 1.53 (1.37 ~ 1.71) *** | 1.80 (1.62 ~ 2.00) *** | 1.43(1.25 ~ 1.64) *** | 1.65 (1.45 ~ 1.88) *** | 1.65 (1.47 ~ 1.85) *** | 1.59 (1.38 ~ 1.84) *** |
| SUA Per 50 unit | 1.16 (1.11 ~ 1.21) *** | 1.15 (1.10 ~ 1.20) *** | 1.14 (1.08 ~ 1.20) *** | 1.14 (1.08 ~ 1.20) *** | 1.21 (1.16 ~ 1.27) *** | 1.15 (1.08 ~ 1.22) *** |
| TyG≤8.89 and Non-HUA | 1 (Reference) | 1 (Reference) | 1 (Reference) | 1 (Reference) | 1 (Reference) | 1 (Reference) |
| TyG ≤ 8.89 and HUA | 1.64 (1.31 ~2.04) *** | 1.53 (1.22 ~1.92) *** | 1.48 (1.14 ~ 1.92) * | 1.59 (1.22 ~ 2.09) *** | 1.80 (1.44 ~ 2.26) *** | 1.59 (1.20 ~ 2.10) *** |
| TyG>8.89 and Non-HUA | 1.48 (1.22 ~ 1.80) *** | 1.81 (1.51 ~ 2.19) *** | 1.43 (1.14 ~ 1.79) * | 1.64 (1.31 ~ 2.07) *** | 1.59 (1.29 ~ 1.96) *** | 1.52 (1.18 ~ 1.96) *** |
| TyG > 8.89 and HUA | 2.31 (1.89 ~ 2.83) *** | 2.73 (2.24 ~ 3.32) *** | 2.29 (1.18 ~ 2.90) *** | 2.36 (1.84 ~ 3.02) *** | 2.76 (2.24 ~ 3.39) *** | 2.29 (1.76 ~ 2.98) *** |

Analysis 1: Base on model2, further adjust lipid-lowering, hypoglycemic, anti- hypertensive. 8939 participants, including 1164 participants with advanced CKM.

Analysis 2: Excluding participants with eGFR of < 60 mL/min/1.73m^2^ (n = 173), 8766 participants remained, including 1027 participants with advanced CKM.

Analysis 3: Excluding participants with eGFR of < 60 mL/min/1.73m^2^ or UACR ≥30(n = 1123), 7816 participants remained, including 676 participants with advanced CKM.

Analysis 4: Excluding participants receiving anti- hypertensive treatment at admission (n = 1378), 7561 participants remained, including 618 participants with advanced CKM.

Analysis 5: Excluding participants receiving lipid-lowering or hypoglycemic treatment at admission (n = 683), 8256 participants remained, including 902 participants with advanced CKM.

Analysis 6: Excluding participants receiving lipid-lowering or hypoglycemic or anti-hypertensive treatment at admission (n = 1764), 7175 participants remained, including 531 participants with advanced CKM.

Adjusted for age, sex, ethnicity, educational level, marital status, smoking status, alcohol drinking, physical activity, daytime napping, and nighttime sleep duration.

TyG index, triglyceride-glucose index; SUA, serum uric acid; HUA, hyperuricemia; CKM, cardiovascular-kidney-metabolic.

* *P* < 0.05

*** *P* < 0.001

Among female‑only participants, both continuous and categorical TyG index showed significant positive associations with advanced CKM syndrome. In the multivariable‑adjusted Model 2, per‑unit increase in TyG index was associated with higher odds of advanced CKM (OR 1.87, 95%CI 1.62‑2.16, *P*<0.001). For continuous SUA, each 50 μmol/L increment was associated with 27% higher odds of advanced CKM (OR 1.27, 95%CI 1.20‑1.35, *P*<0.001). After further adjustment for menopausal status in Model 3, the magnitude of ORs remained nearly unchanged. For combined TyG‑HUA subgroups, participants with both elevated TyG index and HUA demonstrated the greatest risk for advanced CKM (Model 3, OR 3.81, 95%CI 2.96‑4.92, *P*<0.001), relative to women with TyG ≤8.89 and non‑HUA. These female‑specific sensitivity analyses suggested that the main findings were robust after accounting for menopausal status.

**Table S5.** **Associations of TyG index and serum uric acid with advanced CKM syndrome among female participants only.**

| Variables | Model1 | |  | Model2 | | Model3 | | |
| --- | --- | --- | --- | --- | --- | --- | --- | --- |
|  | OR (95%CI) | *P* |  | OR (95%CI) | *P* | | OR (95%CI) | *P* |
| TyG Per unit | 1.977 (1.747 ~ 2.238) | <0.001 |  | 1.871(1.621 ~ 2.160) | <0.001 | | 1.872(1.621 ~ 2.161) | <0.001 |
| SUA Per 50 unit | 1.334 (1.265 ~ 1.406) | <0.001 |  | 1.270 (1.196 ~ 1.347) | <0.001 | | 1.270 (1.196 ~ 1.347) | <0.001 |
| TyG≤8.89 and Non-HUA | 1.00 (Reference) |  |  | 1.00 (Reference) |  | | 1.00 (Reference) |  |
| TyG ≤ 8.89 and HUA | 2.555 (1.963 ~ 3.3325) | <0.001 |  | 2.059 (1.537 ~ 2.759) | <0.001 | | 2.060 (1.537 ~ 2.760) | <0.001 |
| TyG>8.89 and Non-HUA | 2.298 (1.817 ~ 2.907) | <0.001 |  | 1.979 (1.530 ~ 2.561) | <0.001 | | 1.980 (1.530 ~ 2.561) | <0.001 |
| TyG > 8.89 and HUA | 4.563 (3.636 ~ 5.728) | <0.001 |  | 3.812 (2.956 ~ 4.915) | <0.001 | | 3.814 (2.957 ~ 4.920) | <0.001 |

Model1: Crude

Model2: Adjust: age, ethnicity, educational level, marital status, smoking status, alcohol drinking, physical activity, daytime napping, and nighttime sleep duration.

Model3: Base on model2, further adjust menopausal status.

After excluding men participants, 5867 participants remained, including 594 participants with advanced CKM. TyG index was analysed both as a continuous variable (per‑unit increment) and as a dichotomous variable (cut‑off value: 8.89). Serum uric acid (SUA) was modelled as a continuous variable per 50 μmol/L increment. Odds ratios and 95% confidence intervals are presented with three decimal places to display minor numerical differences between the two female‑only models. Combined TyG‑HUA categories: TyG ≤8.89 and non‑HUA served as the reference group. OR, odds ratio; CI, confidence interval; TyG, triglyceride‑glucose index; SUA, serum uric acid; HUA, hyperuricemia.

**Table S6. Multicollinearity diagnostics for covariates in Model 2**

| Variable | VIF / Adjusted GVIF^1/(2⋅df)^ |
| --- | --- |
| Age | **1.115** |
| Sex | **1.286** |
| Ethnicity | **1.001** |
| Education | **1.046** |
| Marital status | **1.004** |
| Smoking status | **1.105** |
| Alcohol consumption | **1.052** |
| Night‑time sleep duration | **1.003** |
| Daytime napping | **1.035** |

Note: Continuous variables are reported with standard variance‑inflation factor (VIF). For categorical variables, adjusted generalized variance‑inflation factor GVIF^1/(2⋅df)^ is presented. Values < 2.0 indicate negligible multicollinearity.
